# Virtual Reality Therapy Takes Greenspace to Older Nursing Home Residents: A Pilot Study

**DOI:** 10.1093/geroni/igaa057.3319

**Published:** 2020-12-16

**Authors:** Jody Blankenship

**Affiliations:** University of Missouri - Columbia, Columbia, Missouri, United States

## Abstract

Nature-based virtual reality (VR) interventions can offer a safe adjunct approach for managing depression and anxiety, but applications of this innovative technology have not been studied in the nursing home (NH) setting. Pleasant events like those generated with VR may provide a psychological time-out from the ongoing stress associated with living in an NH, and thereby, may facilitate adaptive coping. This study, conducted between January and February of 2020, aimed to pilot test the effects of ViRT-Ta-GO: Virtual Reality Therapy Takes Greenspace to Older NH residents on the severity of depressive and anxiety symptoms among NH residents aged 60 years and older and obtain estimates of effect sizes on outcome measures. Using a quasi-experimental, one-group, pretest-posttest design, nine cognitively intact NH residents (Mage = 81, SD = 10.23) were recruited from two Pennsylvania NHs. The outcome measures included the Patient Health Questionnaire-9, Geriatric Anxiety Inventory, and 10 open-ended questions. Residents participated individually in three 10-minute VR sessions per week for four weeks. There were no adverse effects. Participant attrition and self-report indicated that ViRT-Ta-GO was feasible and acceptable. At posttest, a Wilcoxon signed rank test showed a significant decrease in anxiety symptoms (z = -2.02, p = .04) with a large effect size (r = -.48). Nonsignificant trend improvements in depression were observed (p = .08), pretest to posttest. The 4-week VR intervention was effective in reducing symptoms of anxiety. Preliminary findings demonstrate utility within an NH population, suggesting it will be advantageous to evaluate ViRT-Ta-GO through controlled clinical trials.

